# Uncharted territories in the discovery of antifungal and antivirulence natural products from bacteria

**DOI:** 10.1016/j.csbj.2021.02.003

**Published:** 2021-02-11

**Authors:** Raghav Vij, Bernhard Hube, Sascha Brunke

**Affiliations:** a Department of Microbial Pathogenicity Mechanisms, Leibniz Institute for Natural Product Research and Infection Biology – Hans Knoell Institute Jena (HKI), Germany; b Institute of Microbiology, Friedrich Schiller University, Jena, Germany

**Keywords:** Antifungal, Antivirulence, Natural products, Fungal pathogens, Biosynthetic gene clusters

## Abstract

Many fungi can cause deadly diseases in humans, and nearly every human will suffer from some kind of fungal infection in their lives. Only few antifungals are available, and some of these fail to treat intrinsically resistant species and the ever-increasing number of fungal strains that have acquired resistance. In nature, bacteria and fungi display versatile interactions that range from friendly co-existence to predation. The first antifungal drugs, nystatin and amphotericin B, were discovered in bacteria as mediators of such interactions, and bacteria continue to be an important source of antifungals. To learn more about the ecological bacterial-fungal interactions that drive the evolution of natural products and exploit them, we need to identify environments where such interactions are pronounced, and diverse. Here, we systematically analyze historic and recent developments in this field to identify potentially under-investigated niches and resources. We also discuss alternative strategies to treat fungal infections by utilizing the antagonistic potential of bacteria to target fungal stress pathways and virulence factors, and thereby suppress the evolution of antifungal resistance.

## 1 Introduction

Of the myriad of fungal species which form critical components of our ecosystem, nearly 600 are known to infect humans [1]. Amongst these, *Candida*, *Aspergillus,* and *Cryptococcus* species can be associated with high morbidity and mortality in patients, particularly such with an impaired immune system. An increase in the population at risk of invasive fungal infections, coupled with emerging antifungal resistance renders pathogenic fungi an eminent threat to public health [2], [3], [4]. Some opportunistic fungi like *Candida auris* and *Lomentospora prolificans* are naturally resistant to many antimycotics [5], [6]. Other species, like *C. glabrata*, show a high intrinsic resistance to specific antifungals like azoles, and some further species, including *C. albicans*, can acquire resistance to antifungals upon exposure, for instance during prophylactic treatments [7]. Yet, there are only a few classes of antifungal drug classes approved and available for treatment of invasive infections caused by a broad spectrum of pathogenic fungi. Altogether, these factors have contributed to a rise in the incidence of infections by drug-resistant fungal strains [8], and call for urgent action in the search for new antifungal therapeutics with distinct mechanisms of action [9].

Many natural products with antimicrobial activities have been identified among microbial natural products. In the ecological context, these compounds defend microbial competitors during interaction of different bacterial and fungal species. In this article, we will review some recent advances in the investigation of bacterial and fungal interaction, and the potential to exploit inter-kingdom molecular communication to develop novel antifungal therapies.

Even before humankind identified microbes as the cause of infectious disease, molds were reportedly used to treat sores and wounds susceptible to bacterial infections in ancient Egypt, China, Greece, and the Roman Empire [10]. As the scientific community began a systematic search for antimicrobial agents, pioneers in microbiology, including John Lister, described the potential of discovering antibiotics by exploiting the interactions between bacteria and fungi [11]. In 1928, these efforts culminated in a paradigm-shifting discovery when Alexander Fleming serendipitously observed that a mold contaminant, later identified as *Penicillium notatum*, was able to lyse colonies of the bacterium *Staphylococcus aureus* [12]. Later, the chemists Chain and Florey worked together to isolate the antimicrobial agent Penicillin. Since then, scientists have frequently looked for natural products secreted by microorganisms to identify novel compounds and scaffolds which can be used to treat maladies ranging from cancer to fungal infections (reviewed in [13], [14]).

The notable contributions of Elizabeth Lee Hazen and Rachel Brown as pioneering women in science (see [15] for a biography) led to the development of the first antifungal drug to be prescribed to humans that was significantly less toxic than previous substances. Their collaboration involved a systematic attempt to survey the soil for organisms that inhibit the growth of deadly fungi, the subsequent isolation of active compounds from crude extracts, followed by an evaluation of toxicity for the promising compounds in model organisms. This approach led to the discovery of the polyene antifungal nystatin, that is secreted by *Streptomyces noursei* [16]. Polyenes bind to ergosterol, an essential sterol in the fungal cell membrane, and kill fungal cells through mechanisms briefly discussed later in this review. Amphotericin B (AmB), secreted by *S. nodosus* [17], is another prominent antifungal polyene that acts on the fungal membrane in a similar manner. While AmB is a potent antifungal with broad-spectrum activity and remains essential for treating invasive fungal infections, polyenes also bind to cholesterol in mammalian membranes and thereby often exerts toxic side effects. Synthetic and semi-synthetic drugs like azoles and echinocandins that target the biosynthesis of fungal cell membranes and the cell-wall, respectively, have emerged as less toxic and frequently prescribed alternatives.

There are only few antifungals in development. Notable compounds in the antifungal pipeline broadly fall into two categories: compounds that have novel mechanisms of action and those that are reformulations or modifications of existing and well-established drug classes (reviewed in [18], [19]). Promising synthetic compounds that fall into the latter category include the azoles BB2603, PC945, VT-1161, and VT-1598. Similarly, rezafungin [20] and SCY-078 [21] are echinocandins, synthetic derivatives of glucan synthesis inhibitors that are secreted naturally by certain fungi. APX001, Olorfim and VL-2397 are novel drugs that inhibit fungal growth by targeting diverse cellular processes. Olorfim inhibits nucleotide biosynthesis [22], while VL-2397 chelates iron that is essential for fungal survival in the host and is secreted by the fungus *Acremonium persicinum* [23], and APX001 (also called fosmanogepix) targets GPI anchors on the cell wall of fungi (reviewed in [24]). The repurposing of the synthetic anti-depressant sertraline for treating cryptococcal meningitis is currently being explored, as it was found to perturb fungal protein translation and significantly lower the fungal burden in the murine brain [25]. Nikkomycin Z is one amongst the very few bacterial natural products in the antifungal pipeline, and highlights the potential for discovery of compounds from bacteria that target distinct mechanisms. The compound, first isolated from *S. tendae* [26], inhibits the biosynthesis of chitin, a critical component of the fungal cell wall. Nikkomycin Z has shown promising success during early clinical trials (phase I) against coccidioidomycosis [27]. Further trials were terminated due to a lack of funding [28].

By theoretical estimates there are approximately 5 million species of fungi [29], [30], and 1 trillion species of bacteria [31], [32], although these estimates remain controversial. Given these numbers, in the diverse ecological niches that bacteria and fungi co-inhabit virtually infinite combinations of interactions are possible. Bacteria and fungi can influence each other’s physical environment, growth, and morphology. They can cooperate with each other, be dependent on one another and/or have an antagonistic relationship. These interactions can have a significant effect on other organisms, including plants and humans. The diversity of their interactions, as well as their effect on human health have been exhaustively reviewed in [33], [34], [35], [36], [37].

Natural products are primarily non-essential substances biosynthesized by bacteria, fungi or plants which are often bioactive and can play a crucial role in ecological interactions as communication signals or chemical weapons for predation or as defense against it. Molecules with antifungal activity are usually natural products, generally produced by bacteria *via* biosynthetic pathways that are encoded by complex biosynthetic gene clusters (BGCs).

For this review, we used systematic and computational approaches [38] to filter the nearly 6,500 papers in the literature on antifungals derived from bacteria and tried to define trends. The results from an NCBI query, “antifungal[mesh] AND bacteria[mesh] AND journal article[publication type]”, indicated a notable increase of interest in the field over the last decades. This raises the crucial question: Why has this large body of literature and intense research resulted in the discovery of only one class of approved antifungals, polyenes, from bacteria?

Keeping aside the enormous challenges of developing new antifungal agents for use in patients [9], [39], in this review, we will discuss the rationale for studying the diversity, and the complexity, of bacterial-fungal interaction as a source of inspiration for antimycotic drug discovery.

### 1.1 Uncharted territories in the prokaryote kingdom

**Fig. 1:**
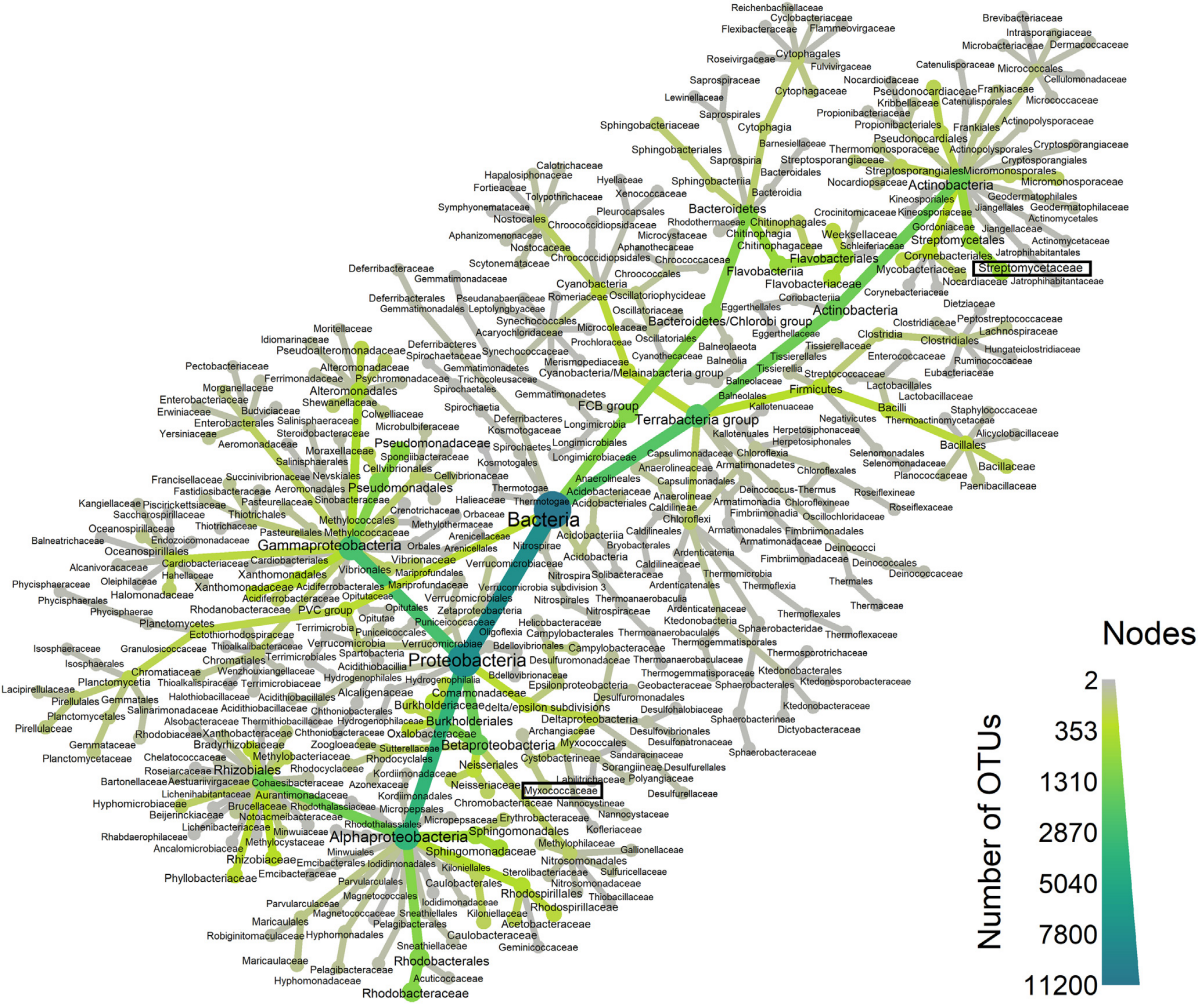
Heat tree depicting the taxonomic distribution of bacterial species that were linked to antifungal research by a PubMed literature search. Each node represents a taxonomic unit, down to families at the leaves. The color and the size of each node indicate the number of species with publications on the topic of antifungal compounds, summed up as operational taxonomic unit (OTUs). For example, Streptomycetaceae are well explored in the literature for their antifungal property with publications on many species, while Myxococcaceae may be represent a yet untapped source for novel drugs (highlighted here with black boxes).

To get a rudimentary estimate of the taxonomic breadth of bacterial species covered in our literature search of bacteria-derived antifungals, we linked our NCBI PubMed query to information from the NCBI Taxonomy database, using the “rentrez” package in R [40]. After removing the ambiguous taxonomy information and selecting only bacterial species, we plotted a heat tree of bacterial families linked to our query (Fig. 1) using the packages “taxa” and “metacoder” [41], [42]. In this tree, the labeled nodes represent bacterial taxomic units, down to families at the leaves, for which we found literature on antifungal activities. Their relative distance corresponds to the degree of taxonomic relationship, and the color and size depicts the number of species for which literature entries exist in PubMed. The tree thereby essentially illustrates the taxonomic distribution of bacteria which have been connected to antifungal research in the past. The wide-spread branching of the heat tree indicates that there is interest and potential for antifungal compounds widely across the bacteria kingdom, but clear hot-spots are visible in certain branches (Fig. 1).

There are two factors that contribute to this unequal distribution of bacterial species abundance in the heat tree of scientific attention (Fig. 1). One is that that there are large variations in the diversity of BGCs among prokaryotes [43]. Some clades intrinsically have a higher genomic potential to synthesize natural products, and their ecological interactions drive the evolution of molecules that allow them to adapt to their environment [43], [44], [45]. A recent study revealed that bacteria found in soils from similar biomes are likely to secrete similar molecules [44], [46], [47], and the biome, e.g. arid *vs* swamp lands, can strongly influence the biosynthetic potential. In the arid soils for example, a high diversity was found that was likely driven by the high abundance of actinobacteria [46]. Within the actinobacteria, certain families like Streptomycetales are already sources of important antifungals like AmB, nystatin and nikkomycin Z, and are known for their abundant and genetically very diverse BGCs [48], rendering them promising model organisms. Antibiotic production is regulated during their life-cycle to defend the sessile Streptomycetales when they release molecules that attract motile prey (reviewed in [49], [50]). Streptomycetales are not only found in soil, but in diverse ecological niches with different competitors, including fungi. Therefore, it is not surprising that the family Streptomycetales presents a densely populated node in the heat tree (Fig. 1). In contrast, in other clades like the Corynebacteriales, the BGCs are generally conserved, and the members seem to have undergone genomic reduction [51]. Species within this clade include human microbiome strains and mycobacteria, which may have conserved BGCs adapted to survival in the host ([48], reviewed in [154]). This decreased genomic potential is likely the reason why the associated nodes are comparatively less dense (Fig. 1).

The second factor for unexpectedly less dense branches in the tree are the clades that have diverse BGCs, but these have not been exhaustively explored. For instance, in our literature analysis, we found that only a few studies described antifungal molecules from Myxococcales species (Fig. 1). Myxobacteria are a fascinating group of bacteria, defined by their intense social interactions, and their ability to form multicellular fruiting bodies when they are under stress (reviewed in [52]). Presumably because of their social lifestyle, Myxobacteria have evolved various mechanisms to communicate with each other, and with other species in their habitat. They are adept in outcompeting other microbes to conquer common resources, and some even predate on other microbes (reviewed in [53], [54]). To prey on fungi and bacteria, myxobacteria rely on a combination of cell wall degrading enzymes, and notably, an array of antibiotic secondary metabolites [55], [56], [57], [58]. *In vitro*, diverse myxobacteria can prey on *C. albicans* [59], and promising novel antifungal molecules, like ambruticin, have been isolated from them [60], [61]. A study by Hoffman *et al* provided an insightful snapshot into the metabolites secreted by nearly ~2300 myxobacteria, and predicted that it is more likely to find unique metabolite profiles in more distantly related myxobacterial genera [62]. This provides a rationale and motivates a deeper exploration of new genera during the search for novel antifungal compounds [63].

However, the observations made by field microbiologists in the 1960s [64] are relevant even today, as a vast majority of bacteria remain unculturable or grow very slowly under laboratory conditions. This phenomenon has been described as “the great plate anomaly” [65]. Through advances in the isolation and culturing of new bacteria [66], [67], [68], [69], [70], and metagenomic approaches of expressing genes mined from unculturable bacteria in model organisms (reviewed in [71]), it is now possible to discover new metabolites from yet untapped bacteria. In the case of slow growing myxobacteria, genetic engineering approaches have facilitated the expression of myxobacterial BGCs in model organisms like *Pseudomonas putid*a, enabling the isolation of novel antibiotic gene products [72], [73]. Finally, given the interest in new myxobacterial species [62], 16s ribosome sequencing of viable but non-culturable isolates has provided insights into their diversity [74], [75], [76], [77]. Transferring the nutrient requirements of related culturable bacteria, and through the development of metagenomic tools, researchers hope to exploit the biosynthetic potential of suborders like Sorangiineae soon [78], [79].

A broad variability in the BGC diversity of bacterial clades has been observed in many previous studies, but there seems to be no clear consensus in the field so far whether phylogeny and biosynthetic potential correlate [43], [51], [62], and thus, whether it is more promising to search for new substances taxonomically distant from known producers, or close by. A heat tree like Fig. 1 can be used as a kind of map that shows the known clades of well-investigated producers like actinobacteria, and the uncharted territories which contain islands of prokaryote families with antifungals awaiting their discovery.

### 1.2 A diverse and hidden arsenal of natural products from bacteria

A closer look at the synthesis of AmB by the actinobacterium, *S. nodosus* shows that some bacteria invest immense efforts to synthesize and secrete small molecules that specifically affect fungal competitors*.* Polyketide synthase (PKS) modular enzymes encoded by six large PKS genes in the AmB gene cluster facilitate specific additions and substitutions to tailor the macrolide backbone of AmB. Two cytochrome P450 enzymes modify the precursor molecule, and several enzymes synthesize and attach a mycosamine moiety that is essential for binding to ergosterol [80], [81]. The molecule is transported by two ABC transporter proteins encoded in the gene cluster, and notably, this complex biochemical pathway is orchestrated by several regulatory factors [82]. The resultant molecule AmB has the exact configuration required to bind and sequester ergosterol, an essential component of the fungal cell membrane [83], [84], which permeabilizes it to causes rapid efflux of ions from the fungal cytosol [85], [86]. The combinatorial effects of AmB lead to rapid fungal cell death, which makes it an important antifungal agent to this day. *S. nodosus* has even more to offer, as its AmB gene cluster also synthesizes amphotericin A, which differs from AmB only by a single bond in place of a double bond [17], [87]. It is postulated that the kinetic competition between different modules of PKS assembly line lead to the formation of both, amphotericin A and B [80].

As hinted at by the two forms of amphotericin, bacteria use several mechanisms to diversify their repertoire of natural products (reviewed in [88], [89]). The organization of genes into BGCs, and the modular nature of enzyme complexes like PKS and non-ribosomal peptide synthetases (NRPS) offer an intrinsic versatility. Entire gene clusters can be horizontally transferred from one species to another. Under selection pressure, or due to genetic drift genes in BGCs undergo substitutions, deletions, rearrangements, and duplications, that result in the synthesis of molecules with different properties and/or functions [90]. For instance, across the actinobacterium clade, there is evidence of diversification of the polyene BGC, particularly at the acetyl-transferase domain, that has led to the synthesis of structurally distinguishable molecules like nystatin, amphotericin, pimaricin, and selvamicin [80], [90], [91], [92], [93]. This diversity can be exploited in drug discovery: The structural differences between selvamicin and nystatin result in significant shifts of properties, such as solubility or the mechanism of action [91].

In 2002, the complete genome sequence of the antibiotic producer, *S. coelicolor* A3(2) was published [94]. An incongruity was observed when the whole genomic sequence of the bacterium was examined and researchers noticed that while there were nearly ≈23 BGCs, only a dozen natural products were produced or had been isolated from the supernatant of lab cultures. The discovery of “cryptic” or “silent” gene clusters across the bacterial (and fungal) kingdom revealed that microbes have far greater genomic potential for the synthesis of natural products than was previously recognized. Sophisticated computational methods have kept pace with the ever-expanding repositories of bacterial genome sequences. Genome mining tools are able to predict the biosynthetic potential of species, and identify promising gene clusters that maybe hidden (reviewed in [95]). For example, a recent metagenomics study described the high biosynthetic potential from unexplored clades in the soil, including unculturable and previously unknown Acidobacteria and species of the candidate phylum Rokubacteria (Fig. 1) [96]. Metatransciptomics showed that the expression of the BGCs in these bacteria varied greatly over time, and was influenced by the presence of substrates like glucose, methanol, and water [96].

The natural product biosynthetic pathways in bacteria are complex and energetically expensive, and therefore it makes sense if they are not expressed in the absence of microbial interaction partners. In a pioneering study, Schroeckh *et al* found that co-cultures of *Aspergillus fumigatus* and soil actinomycetes activated silent gene clusters in fungi, which led to the synthesis of previously unknown antibacterial metabolites [97]. A similar rationale has also led to the discovery of jagaricin, an antifungal compound secreted by *Janthinobacterium agaricidamnosum* (see family Oxalobacteraceae in heat tree, Fig. 1), a bacterium that causes soft rot disease in button mushrooms, *Agaricus bisporus* [98]. A genome mining approach revealed the potential to secrete antifungal metabolites, but these BCGs were not expressed in standard cultures [99]. Graupner *et. al* cultured the bacterium quasi-naturally on mushroom slices, and thereby induced the expression of the cryptic gene cluster that allowed production of jagaracin, which in turn was detected by imaging mass spectrometry [99]. Notably, jagaricin exhibited a broad spectrum of activity against plant fungal pathogens, making it a promising starting point for a compound that may be useful in agriculture [100].

Several studies that considered ecological interactions as the driver of evolution of BGCs have advanced the discovery of antifungals. Certain bacteria, like species in the Pseudonocardiaceae and Streptomycetaceae families (see noticeably dense nodes, Fig. 1), protect ants from fungal infections [101]. Based on this rationale, Haeder *et al.* sought to isolate antifungal molecules from bacteria associated with leaf-cutter ant colonies [102]. The bacteria were found to synthesize candicidin, a polyene macrolide, that had potent activity against the fungal ant pathogen *Escovopsis* sp., but only little effect on the growth of symbiotic fungi [102]. Isolation of bacteria from ant colonies also led to the discovery of several other antifungal molecules, including selvamicin and dentigerumycin [91], [103], [104], [105]. Clearly, ecological niches where fungi and bacteria closely interact, like the soil [46], [106], lichen [107], and insect microbiomes [108], [109], are promising sources of antifungal molecules (reviewed in [37]). A combination of metabolomic, transcriptomic, and metagenomic data therefore is a promising avenue to compare networks of microbial interactions in different biomes (reviewed in [110]) – and in biomes that are enriched in bacteria-fungi interactions, future discoveries of new antifungal compounds are very likely.

### 1.3 Bacterial natural products to target fungal stress responses

Conventionally, our rationale in the search for antifungal drugs is limited to screening for molecules that have potent fungicidal or fungistatic activity. The goal therein is to discover compounds so potent, that they decimate entire populations of pathogens during infection. Realistically, however, through various mechanisms, fungi develop resistance (reviewed in [111]) or gain tolerance in the host (reviewed in [112]). One measure to oppose that are combination therapies, the gold standard of anti-infective therapy is the combinatorial treatment against tuberculosis that targets up to four distinct cellular processes to kill the bacteria and prevent the emergence of resistance. However, most antifungal drugs in use today only target single cellular processes, like cell wall or membrane synthesis and repair, and development of resistance is accordingly common.

Bacteria in interaction with fungi should face the same problem of their targets becoming resistant, and it seems likely that microbes have evolved strategies to lower the rate of evolutionary resistance development in fungi. The numerous BGCs bacteria often encode may be one of them, as the synthesized plethora of molecules may sometimes act synergistically and target diverse cellular processes, to give the organism a competitive advantage (discussed in [113], [114]). It may be worthwhile, therefore, to again turn to microbial interactions for the discovery of compounds that suppress the evolution of resistance.

Fk506 [115] and geldanamycin [116] were discovered and isolated from actinobacteria, while screening for drugs with antimicrobial properties. Later studies found that they target cellular stress response conserved across eukaryotes, and since then they have been studied extensively for their immunosuppressive (FK506) and anti-cancer (geldanamycin) potential in humans. The need for antifungal drugs with novel mechanisms has led researchers to reexamine and repurpose these drugs for their antifungal potential.

For example, the immunosuppressant FK506 binds to the protein FKB12, such that the FK506-FKB12 complex inhibits Calcineurin, a phosphatase required for T-cell activation in mammals [117]. In pathogenic fungi like *Cryptococcus neoformans*, *C. albicans*, and *A. fumigatus*, its ortholog was found to be essential for virulence, thermotolerance, and antifungal drug tolerance, making it a promising drug target (reviewed in [118]). Notably, FK506 also acts synergistically with antifungal drugs (reviewed in [119]).

One obvious hindrance in pursuing FK506 and geldanamycin for clinical use is their off-target and detrimental effects on mammalian cells. For instance, the immunosuppressive effects of FK506 could interfere with the patient’s immune response to the fungal infection, and lead to poor outcomes, as was observed in a cryptococcosis murine model [120]. To overcome this, structure–activity based modeling approaches have been used to develop and identify new molecules that specifically target FKBP12 [118], [120], [121], [122] in fungi, but that do not effect mammalian cells (reviewed in [123]). A promising alternative is to use genome mining to identify similar biosynthetic gene clusters in bacteria, and identify fungal specific effector molecules. We may also discover novel molecules with unexpected targets and functions: The immunosuppressants FK506 and rapamycin are structurally related molecules synthesized by a gene clusters of similar origin, and both were discovered as novel antifungals, but they have very different modes of action [90], [124].

Geldanamycin, is a potent and specific Hsp90 inhibitor that was also first isolated from bacteria [116]. Hsp90 is a molecular chaperone that stabilizes proteins like calcineurin that are induced upon exposure to antifungal drugs, and it helps to stabilize fungal cells long enough for them accrue mutations that confer resistance [125], [126], [127]. Combinatorial treatment of fungi with geldanamycin and azoles or echinocandins prevents the rapid selection of antifungal resistance [128]. It remains to be seen whether the “evolution-suppressor” effect of these molecules that affect antifungal tolerance *in vitro* (and in murine models), also affects fungal competitors of the bacteria that secrete them in co-cultures or microbial communities. Bacteria that synthesize geldanamycin and similar molecules have been isolated from habitats ranging from marine worms to soil [116], [129], [130], and we may yet discover molecules that specifically target fungal pathogens of humans or plants, but not human cells.

However, certain mechanisms of antifungal resistance may confer cross resistance to geldanamycin and FK506, particularly mutations that lead to an increase in drug efflux pump activity [120], [126]. It is likely that this phenomenon will extend to compounds discovered by genome mining approaches that search for new bacterial BGCs. Another limitation in genome mining is that we cannot predict novel molecules from previously undescribed and unique gene clusters. Therefore, unbiased screening to discover new compounds from bacteria, possibly in co-cultures with fungi, remains relevant.

### 1.4 Bacterial natural products to disarm fungal virulence

With gathering evidence on the detrimental impact of fungal dysbiosis on health, it becomes also crucial to think beyond the use of broad spectrum antifungals that detrimentally impact the mycobiome ([131], reviewed in [132]). Most fungi that cause disease do so from opportunistic infections, and they use specific virulence factors to damage the host (reviewed in [133]). An alternative to eradication of fungi is therefore to target their virulence factors. If these virulence factors are not essential for survival in the host, there should be no strong selective pressure, and this strategy would largely avoid the rapid development of resistant mutants.

For example, *C. albicans* can switch between yeast and hyphae morphologies, and the hyphal form is associated with host cell invasion, damage and virulence. Therefore, *C. albicans* dimorphism is a notable target for antivirulence drugs (reviewed in [134], [135]). A series of clinical observations from the 1980s, on the co-occurrences of bacteria and fungi in infections, first garnered an interest in the direct impact of such interactions on human health [36], [136]. Following up on one such frequently co-occurring pair, Hogan *et. al* observed that *P. aeruginosa* and *C. albicans* influence each other’s behavior [137], and these interactions affect the outcome of a mixed infection. Later they also discovered 3OC12HSL, a molecule secreted by *P. aeruginosa* that mimics the fungal quorum sensing molecule farnesol, and modulates *C. albicans* morphogenesis, but not growth [138]. Molecules that target other important virulence attributes of *C. albicans,* including biofilm formation and fungal adhesion have also been discovered from bacteria ([139], [140], [141] and reviewed in [142]).

*C. neoformans* and *C. gattii* can evade host defenses by forming a protective polysaccharide capsule, and a black-brown pigment called melanin. *Cryptococcus* species are commonly isolated from trees, soil, water, and pigeon stool, which first led researchers to examine their ecological interactions with bacteria (reviewed in [143]), where they found numerous strains of bacteria from pigeons that inhibited *Cryptococcus* spp. growth. Mayer *et al.* investigated antivirulence effects of environmental isolates of *Bacillus* spp., which were found to inhibit cryptococcal melanin and capsule formation, but did not significantly alter its growth [144]. The strain *Bacillus safensis* also inhibited *C. albicans* filamentation, biofilm formation, and adhesion [144]. Intriguingly, the authors did not find any small molecules secreted by the bacterium that affected virulence of *C. neoformans* [144]. Instead, they found that the bacteria swarm and surround fungal cells, and their antivirulence effect is due to a cell–cell contact mediated degradation of the fungal cell-wall polymer chitin [144]. Other notable antivirulence targets include metalloproteases that allow *C. neoformans* cells to cross the blood brain barrier where they cause fatal meningitis (reviewed in [145]).

To take advantage of the many mechanisms bacteria use to dynamically suppress fungal virulence, there has been considerable interest in screening and identifying bacteria that can be used as live therapeutics. Numerous studies have indicated that *Lactobacillus* spp. can decrease mucosal *Candida* infections (reviewed in [146]). Lactobacilli affect *C. albicans* in a contact-dependent manner, and secrete molecules ranging from hydrogen peroxide to proteins that degrade chitin, which then in combination affect hyphae and biofilm formation [147], [148]. In the presence of *L. rhamnosus*, host epithelial cells associated with *C. albicans* cells are shed and at least some seem to die via apoptosis, which allows for the renewal and maintenance of the epithelial barrier [149].

How the diversity and composition of fungal and bacterial species that constitute our microbiome effect human health is just beginning to be understood. Metabolomics and metagenomics of our human microbiome will help us map out microbial interaction networks [150], [151], and lead to identification of species or metabolites that can be used to control fungal infections [152], [153].

## 2 Summary and outlook

The high morbidity due to drug-resistant fungal species and strains underscores the need to develop new antifungal agents. Drugs like AmB and nystatin that were discovered from bacteria decades ago, and continue to be used in clinical practice, still motivate natural product discovery to look towards bacteria as sources of novel antifungals. Some bacterial clades, like Actinobacteria, have been studied extensively as sources of antifungals, but other clades with immense biosynthetic potential, like Myxobacteria, remain largely unexplored (Fig. 1). We have to intensify our searches among novel bacteria from ecological niches where antifungal molecules are likely to confer a fitness advantage. Additionally, antifungals are just one of the many molecular mediators that manipulate diverse cellular process in fungi. For example, molecules that target fungal stress responses can be used in combination therapy to suppress the evolution of drug resistance. With a deeper understanding of the multitude of bacteria-fungi interactions, we may also discover molecules or species that specifically suppress fungal virulence.

## CRediT authorship contribution statement

**Raghav Vij:** Conceptualization, Software, Writing - original draft, Visualization. **Bernhard Hube:** Conceptualization, Writing - review & editing, Supervision, Funding acquisition. **Sascha Brunke:** Conceptualization, Writing - review & editing, Supervision, Funding acquisition.

## Declaration of Competing Interest

The authors declare that they have no known competing financial interests or personal relationships that could have appeared to influence the work reported in this paper.
